# Foamy Virus Biology and Its Application for Vector Development

**DOI:** 10.3390/v3050561

**Published:** 2011-05-11

**Authors:** Dirk Lindemann, Axel Rethwilm

**Affiliations:** 1 Institut für Virologie, Medizinische Fakultät “Carl Gustav Carus”, Technische Universität Dresden, Fetscherstr. 74, 01307 Dresden, Germany; 2 DFG-Center for Regenerative Therapies Dresden (CRTD)—Cluster of Excellence, Biotechnology Center, Technische Universität Dresden, Fetscherstr. 74, 01307 Dresden, Germany; 3 Institut für Virologie und Immunbiologie, Universität Würzburg, 97078 Würzburg, Germany; E-Mail: axel.rethwilm@mail.uni-wuerzburg.de

**Keywords:** Foamyviruses, retroviral vectors, LAD, Fanconi Anemia

## Abstract

Spuma- or foamy viruses (FV), endemic in most non-human primates, cats, cattle and horses, comprise a special type of retrovirus that has developed a replication strategy combining features of both retroviruses and hepadnaviruses. Unique features of FVs include an apparent apathogenicity in natural hosts as well as zoonotically infected humans, a reverse transcription of the packaged viral RNA genome late during viral replication resulting in an infectious DNA genome in released FV particles and a special particle release strategy depending capsid and glycoprotein coexpression and specific interaction between both components. In addition, particular features with respect to the integration profile into the host genomic DNA discriminate FV from orthoretroviruses. It appears that some inherent properties of FV vectors set them favorably apart from orthoretroviral vectors and ask for additional basic research on the viruses as well as on the application in Gene Therapy. This review will summarize the current knowledge of FV biology and the development as a gene transfer system.

## 1. Introduction

Spuma- or foamy viruses (FVs) have co-evolved with their natural hosts and are endemic to most non-human primates, cats, cattle and horses [1,2]. Man is not a natural host for FVs but can be infected through zoonosis [3,4]. A hallmark of FVs is their apparent apathogenicity in natural hosts as well as in infected humans. This is in stark contrast to their highly cytopathic nature *in vitro*, where infection ultimately results in the death of most target cells due to syncytia formation and vacuolization.

Research on FVs during the last two decades has revealed a very special replication strategy of these viruses, combining features of both retroviruses and hepadnaviruses [5]. As a result, a couple of years ago the family of retroviridae was reorganized [6]. FVs now constitute the single genus of spumaviruses in the *Spumaretrovirinae* subfamily of retroviruses and are set apart from all other retroviral genera that make up the subfamily of the *Orthoretrovirinae*.

The best-studied FV species is the Prototype FV (PFV), which for many years was known as human FV (HFV). It was first described in the early 70s of the last century after isolation from a nasopharyngal carcinoma from a Kenyan patient [7]. However, its high sequence homology to FVs from chimpanzee (SFVcpz) and the lack of evidence of “natural” human infections strongly suggests that this virus is derived by a zoonotic transmission from a chimpanzee [8–11]. Such *trans*-species transmissions appear to occur commonly upon occupational of casual contact of men with non-human primates [4,12].

This review summarizes our current knowledge on the biology of FVs, concentrating predominantly on PFV as well as their development and application as a gene transfer tool.

## 2. The FV Replication Cycle, an Overview

**Figure 1:**
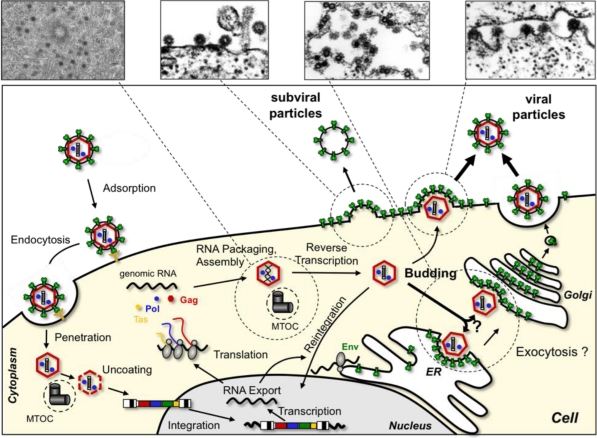
Schematic overview of the spuma- or foamy viruses (FV) replication cycle. Electron micrographs (courtesy of H. Zentgraf and J. Krijnse-Locker, Heidelberg) of different steps of FV particle morphogenesis are shown in the upper panel.

To begin we shall give a brief overview of our current knowledge of the sequential steps of FV replication *in vitro* (Figure 1). FV infection starts with attachment to target cells and binding to an, as yet unknown, but potentially very ubiquitous cellular receptor(s). It is thought that most FV species enter target cells predominantly by receptor-mediated endocytosis and the FV glycoprotein-dependent release of intact capsids into the cytoplasm requires a pH-dependent fusion process [13]. Upon arrival of capsids into the cytoplasm, they seem to dock to dynein motor protein complexes and migrate along microtubules towards the microtubule organizing center (MTOC) where they accumulate [14]. In resting cells, FV capsids can be detected at the MTOC for very long periods and still remain infectious [15]. Further disassembly apparently involves capsid processing by viral and cellular proteases and appears to be cell cycle dependent [16–18]. Nuclear localization of the FV preintegration complex, whose composition has not been characterized in detail, seems to require nuclear membrane breakdown [17,18]. A tethering of FV Gag to chromatin by a specific sequence motif, thereby enhancing viral integration, has been suggested [19]. Upon establishment of the proviral state, expression of FV genes by the cellular transcription machinery is regulated through a viral transactivator utilizing internal and LTR derived promoter elements [20]. Differentially spliced RNAs are exported out of the nucleus, some by a novel CRM1-dependent pathway [21]. FV Gag protein has recently been implicated to contribute to nuclear RNA export as well [22]. FV protein translation of accessory, capsid and enzymatic gene products takes place in the cytoplasm, whereas glycoproteins are translated at the rough endoplasmic reticulum (ER) and targeted to the secretory pathway. FV glycoprotein intracellular transport and cell surface expression are negatively regulated by an ER retrieval signal and ubiquitination [23,24]. FVs follow a B/D morphotype retroviral assembly strategy involving transport of Gag to the MTOC where a preassembly of capsids takes place. Unlike orthoretroviruses, FVs reverse transcribe their packaged RNA genome after capsid assembly in virus-producing cells and those particles containing full-length viral DNA (about 10–20% of total) contribute most to viral infectivity [25–27]. Furthermore, in an *in vitro* system a nuclear reshuttling and reintegration of viral genomes in producer cells was observed [28,29]. Budding and particle release of FVs into the environment is strictly Env-dependent, since FV Gag apparently lacks a membrane-targeting signal typically present in orthoretroviral capsid proteins [30,31]. Therefore, FVs are unable to release virus like particles (VLPs) in an orthoretroviral fashion. Virion budding of most FV species is observed predominantly at putative intracellular membranes of ill-defined origin (e.g., ER, Golgi) but to a limited extent also at the plasma membrane. For release of particles from the infected cells FVs like other viruses exploit the cellular machinery of vacuolar protein sorting (Vps) [32,33]. Unlike other retroviral glycoproteins the FV Env appears to contain structural functions involved in particle formation, which results in the additional release of capsidless subviral particles (SVPs) from FV infected cells, a process that is controlled by posttranslational modification of the glycoprotein [23,33,34].

## 3. Genome Organization and Transcription

**Figure 2:**
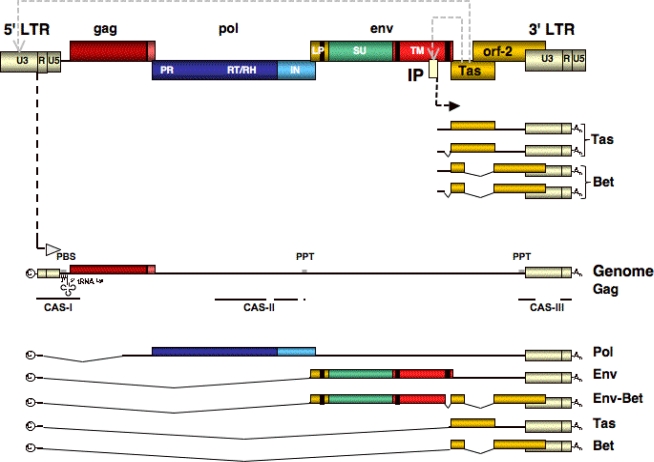
Spuma- or foamy viruses (FV) RNA and DNA genomic organization. Schematic outline of the FV proviral genome (top) and the genomic (middle) and subgenomic transcripts generated by cellular RNA polymerase from long terminal repeat (LTR) and internal promoter (IP). c: cap structure; An: poly-alanine; CAS: *cis*-acting sequence; PPT: poly purine tract: U3: LTR unique 3′ region; R: LTR repeat region; U5: LTR unique 5′ region; PR: protease domain; RT/RH: reverse transcriptase-RNAse H domain; IN: integrase domain; LP: leader peptide domain; SU: surface domain; TM: transmembrane domain.

The typical proviral genome of FVs resembles that of other complex retroviruses (Figure 2). In addition to the canonical open reading frames (ORFs) encoding a group specific antigen (Gag), a polymerase (Pol) and an envelope (Env) protein, flanked by long terminal repeats (LTRs), FVs contain at least two additional ORFs, one extending into the 3′ LTR, that encode proteins with regulatory and immunomodulatory functions.

An unusual feature of FV proviral organization is the presence of an internal promoter (IP) within the *env* ORF, in addition to the typical retroviral promoter located in the U3 region of the 5′ LTR [34]. The IP, which has a low basal activity, drives expression of the accessory genes [35,36]. One of these, Tas (*trans*-activator of spumaviruses), is a potent transcriptional activator [37,38]. The details of FV transcription regulation have still to be worked out but it is known that Tas binds to specific sequences upstream of the IP [39,40]. Through a positive feedback loop Tas thereby increases its own expression. Further accumulation of Tas subsequently leads also to *trans*-activation of the FV LTR promoter by recognition of specific sequence motifs in the U3 region, resulting in transcription of the FV structural genes and genomic RNA [20,39,40]. In comparison to the IP, binding of Tas to the LTR U3 region appears to occur with lower affinity, but higher avidity [20,39,40]. In the absence of Tas, the U3 promoter is virtually transcriptional inactive [38]. By this mechanism FVs appear to regulate the balance between early and late protein expression [21]. However, with the probable exception of bovine FV (BFV) [41–43], the contribution of cellular factors and their interaction with the FV transactivator Tas is largely unknown.

The second, more abundant protein translated from mRNAs originating predominantly from the IP is Bet [44,45]. For a long time no essential function could be attributed to this protein for *in vitro* replication of FVs [46]. It is only in recent years that a function for Bet has evolved as a viral antagonist for the host cells’ innate defense system, by neutralization of cellular APOBEC3 protein functions [47–49].

As a retrovirus FV faces the problem of nuclear export of differentially spliced RNAs. Complex orthoretroviruses express accessory proteins that facilitate export of unspliced and partially spliced viral RNAs by recognition of specific RNA secondary structure elements. For example, the HIV-1 Rev binds within these viral transcripts linking them to the CRM1-dependent cellular RNA export pathway [50,51]. No such function has been reported for any of the FV accessory proteins. In contrast, unspliced transcripts of simple orthoretroviruses appear to harbor specific RNA structural elements (CTE, constitutive transport elements) that directly feed the respective viral RNAs into the cellular NXF1/NXT1 RNA export machinery [52,53]. A very recent report suggests that FVs utilize yet another mechanism, a mixture of both orthoretroviral export strategies. Export of unspliced and single spliced FV structural protein encoding mRNAs appears dependent on the CRM1-dependent RNA export machinery. However, unlike other complex retroviruses crosslinking to CRM1 is mediated by additional cellular proteins and not, as in the case of orthoretroviruses, by a viral protein [21]. Sequence elements within the FV RNAs essential for nuclear export still await their characterization. In addition, a potential role of FV Gag in viral RNA export has been proposed recently, based on a putative nuclear export signal (NES) in the N-terminal domain of the protein [22].

Another feature distinguishing FVs from orthoretroviruses is the expression of Pol from a separate singly spliced RNA [54–56]. Thus, FVs unlike all other retroviruses express no Gag-Pol fusion protein [57]. This raises a couple of important questions on the FV replication strategy. Firstly, how do they control the balance between capsid and polymerase protein translation that appears to be critical for proper capsid assembly and virion infectivity of orthoretroviruses? It was reported that cellular FV Pol levels are regulated by an inefficient Pol splice acceptor site located in the *gag* ORF [58]. In addition, FVs seem to tolerate greater differences in the ratios of Gag and Pol proteins than orthoretroviruses, since in-frame Gag-Pol fusion proteins still support the generation of infectious FV particles, whereas in the case of orthoretroviruses this abolishes infectious virus production [59]. Secondly, this unusual Pol biosynthesis as an independent protein also necessitates a Pol particle incorporation mechanism that is different from that of orthoretroviruses. In the case of FVs the full-length viral genomic RNA seems to bridge Gag and Pol proteins during assembly, as RNA and Pol incorporation requires recognition of specific regions within the virus genome by Gag and Pol [60–62]. However, additional protein-protein interactions of both proteins were reported to be required for Pol particle incorporation as well [63]. Interestingly, only the Pol precursor protein, but not the mature cleavage products, appears to be efficiently packaged into assembling FV particles [62,64].

## 4. FV Structural Proteins

**Figure 3:**
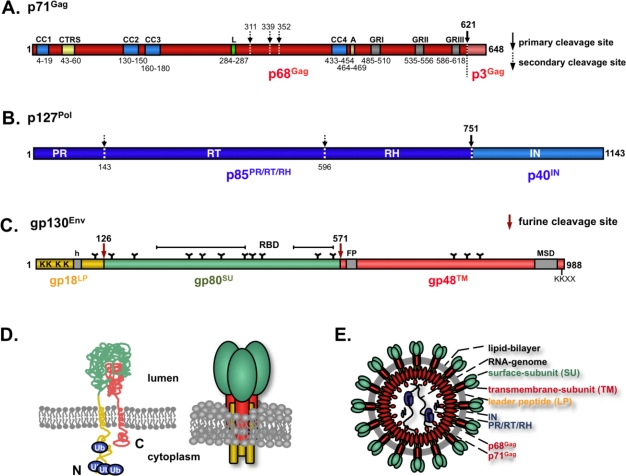
The spuma- or foamy viruses (FV) structural gene products. Schematic of domain organization of Prototype FV (PFV) Gag (**A**), PFV Pol (**B**), and PFV Env (**C**). (**D**) Proposed membrane topology and oligomeric organization of PFV Env. (**E**) Schematic outline of a PFV particle. CC: coiled-coil motif; L: PSAP late-assembly (L)-domain motif; A: YXXLGL assembly domain motif; GR: glycine-arginine rich box; PR: protease domain; RT: reverse transcriptase domain; RH: RNAse H domain; IN: integrase domain; h: hydrophobic domain of the leader peptide (LP); FP: fusion peptide of the transmembrane subunit (TM); MSD: membrane-spanning domain of the TM subunit; N: N-terminus; C: C-terminus.

The genome of FVs encodes the canonical *gag*, *pol* and *env* retroviral structural genes. However, their biosynthesis and functions deviate significantly in several aspects from their orthoretroviral homologues and are summarized here (Figure 3).

## 5. FV Gag

Like all retroviruses, FVs translate a Gag precursor protein that is encoded by the full-length or (pre-) genomic viral RNA. In the case of PFV, this is a protein of 71 kD in size. Efficient FV Gag translation requires an upstream splice donor (SD) site, but not necessarily the original FV SD within the LTR R region [65,66]. Unlike orthoretroviruses the FV Gag undergoes only a limited proteolytic cleavage upon maturation [67]. The typical orthoretroviral processing products matrix (MA), capsid (CA), and nucleocapsid (NC) are not observed during FV Gag biosynthesis. During capsid assembly the FV Gag precursor (PFV p71^Gag^) gets cleaved only once near the C-terminus resulting in a larger (PFV p68^Gag^) and a smaller (PFV p3^Gag^) cleavage product. The viral capsid is composed of the precursor protein and the larger cleavage product at a ratio of 1:1 to 1:4 [68]. Gag precursor cleavage is essential for infectivity but not particle release, since particles containing only Gag precursor are exported but are non-infectious and show incompletely closed capsids in ultrastructural analysis [69,70]. By contrast, particles containing only the larger cleavage product and no precursor display a particle morphology and pattern of release that are indistinguishable from those of the wild type but are about 100-fold less infectious [69]. Some reports suggest that further FV Gag processing by the viral and cellular proteases occurs upon target cell entry and is essential for productive infection, probably by controlling capsid disassembly [16,71].

Several functional domains or motifs have been characterized within the FV Gag protein, although it does not display the typical orthoretroviral MA, CA, and NC subdomain structure. In the first place, PFV Gag harbors four predicted coil-coil (CC) domains [72]. The most N-terminal domain (CC1) seems to be involved in Gag-Env interactions that are essential for particle release (see below). The second coiled-coil domain (CC2) is reported to provide Gag-Gag interaction essential for capsid assembly [73]. It is suggested that the third (CC3) mediates interaction with light chains of dynein motor protein complexes that apparently are exploited by FVs for transport of incoming particles to the MTOC [14]. No function has been assigned to the fourth coiled-coiled domain (CC4). Secondly, a motif with strong homology to the B/D morphotype of retroviruses cytoplasmic targeting and retention signal (CTRS) is thought to be responsible for Gag transport to the MTOC where preassembly of newly generated FV capsid takes place [74–76]. However, unlike Mason-Pfizer monkey virus (MPMV), where specific mutations in the CTRS lead to a conversion from a B/D-type towards a C-type capsid assembly strategy, all currently examined FV CTRS mutations abolish capsid assembly completely and result in a nuclear accumulation of the mutant proteins [77]. Thirdly, like many other viruses FV exploit the cellular Vps machinery, in particular the ESCRT protein complexes, during the late stages of particle release to completely pinch-off from cellular membranes. Therefore, FV Gag proteins harbor an L-domain [32,33]. Experimentally L-domain function has so far only been confirmed for PFV Gag and it has been shown that interaction of a PSAP L-domain motif with cellular TSG101 (a component of ESCRT-I) is essential for efficient particle release [33]. Interestingly, only the primate FV Gag proteins contain PSAP L-domain motifs, suggesting that they all use TSG101 and ESCRT-I for pinch-off. However, the fact that non-primate FVs lack this type of L-domain raises the question as to whether they utilize the Vps machinery as well, potentially by linking Gag to the ESCRT machinery through other L-domain motifs. Fourthly, a conserved YXXLGL motif close to CC4 appears to have a function in proper capsid assembly by influencing capsid morphology and thereby the efficiency of intraparticular reverse transcription similar as the C-terminal domain (CTD) of the CA subunit of HIV (see below) [78]. Fifthly, FV Gag proteins lack the typical Cys-His motifs of orthoretroviral Gag proteins with implicated function in different steps of retroviral replication including genome packaging [79]. Instead, the C-terminus of the larger Gag processing product in all FV species is rich in glycine (G) and arginine (R) residues [80]. In primate FVs these are clustered in three GR-rich boxes (GR-boxes) and are thought to play roles in viral replication similar to those of the orthoretroviral Cys-His boxes [81]. GRI was originally thought to be essential for nucleic acid binding, but two studies failed to confirm this and suggested functions in Pol encapsidation or capsid morphology and reverse transcription [81,82]. An NLS function was initially attributed to GRII being responsible for the transient nuclear targeting of Gag during certain time points of PFV infection [80]. However, the requirement of the NLS for PFV replication, has been questioned and, furthermore, some non-primate FV Gag proteins fail to localize to the nucleus [54,83]. The GRII harbors a chromatin-binding site (CBS) that was proposed to tether incoming capsid to chromatin by histone interactions and facilitate integration [19]. Finally, there may a role for GRII in intra-particular reverse transcription, thereby influencing infectivity of released PFV particles [81]. Furthermore, GRIII, the function of which is not known, may have a similar role in intraparticular reverse transcription [81]. Other peculiarities of FV Gag, such as the highly unusual rareness in lysine residues, were highlighted recently [72,84]. Quite recently a putative NES signal in the N-terminal domain of PFV Gag was reported [22]. Based on this a similar function in nuclear export of unspliced or single spliced viral RNAs was proposed, as described for RSV Gag. However, this data awaits further confirmation.

## 6. FV Pol

Biosynthesis of FV Pol is unusual because of its translation as an independent precursor protein (PFV p127^Pol^) from a separate mRNA and because of its special encapsidation strategy (see above). In addition FV Pol precursor maturation is different from orthoretroviruses. FV Pol is autocatalytically processed into only two subunits, a larger one (PFV p85^PR-RT-RN^) with protease (PR), reverse transcriptase (RT) and RNAseH (RN) enzymatic activities and a smaller one (PFV p40^IN^) with integrase activity. This is in contrast to the standard orthoretroviral processing into PR, RT and IN subunits [67]. Staining of FV infected cells with specific monoclonal antibodies indicated a nuclear localization of both FV Pol molecules [85]. However, a putative NLS signal has only been described for the FV IN subunit [86].

In terms of its biochemical properties FV RT appears to be more active and characterized by higher processivity than, for example, HIV-1 RT, while the *in vitro* fidelity appears to be similar [87–90]. Cell culture experiments, however, showed an unprecedented high fidelity of FV reverse transcription [91].

Retroviral PRs are only active in a dimeric state, which raises the question of how FV PR activity is regulated. Interestingly, recombinant PFV and SFV PR-RT domains are predominantly monomeric in solution, but appear to have some proteolytic activity that is enhanced by high salt conditions [87,92,93]. One publication suggests that recombinant FV PR is able to form transient dimers and therefore escapes detection by traditional methods [92]. Another report proposed that PR dimerization and activity might be regulated at the precursor protein level by the IN oligomerization domains, favoring protein-protein interactions [94]. However, strong evidence has been provided in favor of the former by identifying a nucleic acid motif (PARM for protease-activating RNA motif) that is bound by the Pol precursor protein and regulates PR activity [95]. Thus, as with encapsidating Pol protein that activates PR, experimental evidence exists for both dominating protein-RNA or dominating protein-protein interactions and definite answers to these questions are still open.

The FV IN subunit has attracted a lot of attention recently, as it was the first retroviral IN for which a crystal structure of the full-length subunit in complex with its viral substrate was obtained [96]. In contrast, for orthoretroviral IN subunits only subdomain structures could be solved so far, mainly as a result of the poor solubility of the respective recombinant proteins. The availability of the PFV intasome structure, its susceptibility to HIV-1 IN strand transfer inhibitors such as raltegravir and elvitegravir and its enzymatic efficiency in *in vitro* integration assays using small viral DNA substrates, has opened up possibilities of revealing the key mechanism of retroviral integration and understanding the basics of IN inhibitor action [97–101].

## 7. FV Env

The FV glycoprotein biosynthesis, like FV Pol, deviates significantly from that of other retroviral Env proteins. Like orthoretroviral glycoproteins, it is translated from a spliced mRNA to a precursor protein that is targeted by an N-terminal signal peptide (SP) to the rough ER and thereby inserted into the secretory pathway (Figure 1). However, in contrast to orthoretroviruses the FV SP is not cleaved co-translationally by the cellular signal peptidase complex. Instead, FV Env is translated as a full-length precursor protein, with N-terminal attached SP (termed the leader peptide LP, because it is not a classical SP), central surface (SU) and C-terminal transmembrane (TM) domain, that initially adopts a type III membrane topology with both N- and C-terminus located in the cytoplasm (Figure 3 C and D) [102,103]. FV Env precursor processing into LP (gp18^LP^), SU (gp80^SU^) and TM (gp48^TM^) subunits occurs along its transport to the cell surface and is mediated by furin or furin-like proteases [104,105]. Proteolytic cleavage of SU and TM, but not LP and SU subunits, is essential for viral infectivity [104]. The protein gets heavily N-glycosylated at 14 out of 15 potential sites and two evolutionary conserved sites, one in SU (N8) and one in TM (N13), are important for viral infectivity [106]. In contrast to orthoretroviruses, the LP subunit, probably embedded in tripartite trimeric glycoprotein complexes, is an integral component of the released viral particle (Figure 3E) and plays important additional roles in the replication cycle of FVs (see below) [75,103,107].

Intracellular transport of primate FV Env protein appears to be regulated by two major signals. The first is a C-terminal KKXX dilysine motif that results in retrieval of most Env proteins from early Golgi compartments into the ER in the absence of coexpression of other FV structural proteins [23], though it is not essential for viral replication [108]. In addition, the N-terminal cytoplasmic domain of the LP subunit is posttranslationally modified by ubiquitination at 4 out of 5 lysine residues [24]. Ubiquitination is not essential for viral particle release, but seems to suppress the glycoproteins intrinsic activity to release SVPs, since an ubiquitination deficient mutant shows a more than 50-fold increase in SVP release [24,109]. Interestingly, the domain within Env that is mediating the specific and essential interaction with the capsid during budding also resides in the N-terminus of LP [103,107]. Two evolutionary conserved tryptophane residues (W_10_, W_13_) are essential for this interaction [102,103]. Due to this specific Gag-Env interaction, which cannot be complemented by any other heterologous viral glycoprotein, FV vectors currently cannot be pseudotyped [110].

Although the ubiquitous cellular receptor(s) of FVs still awaits identification a rough characterization of the receptor-binding-domain (RBD) within the PFV Env SU subunit was established, demonstrating that amino acid (aa) 225 to 396 and 484 to 555, including the conserved N-glycosylation site N8, are essential to form the RBD [104]. The FV Env protein is responsible for the extremely broad host range of the viruses. Even evolutionary very distant cells from species like reptiles or birds are permissive for FV Env-mediated vector transduction [111]. Only recently, two cell lines of fish and human origin that appear to be resistant to FV Env-assisted gene transfer and might serve as useful tools to identify the elusive cellular receptor(s) were identified [112].

## 8. FV Egress

Many steps of assembly and morphogenesis of FV particles resemble those of typical B/D-type retroviruses, such as MPMV. These viruses transport their Gag protein translated in the cytoplasm to an intracellular capsid assembly site [79]. Budding of the pre-assembled capsid then takes place unlike C-type retroviruses that assemble their capsid at and bud from cellular membranes simultaneously [79]. FV Gag is transported to the cellular centrosome in a microtubule-dependent manner involving a specific Gag CTRS motif [76]. Accumulation of naked capsids can be observed in ultrastructural analysis [76]. Unique amongst retroviruses are several features of the FV egress process. First, is the dependence of FV capsids on coexpression of the cognate Env protein for subsequent steps of viral particle release [110]. FV capsids lack a membrane targeting signal resulting in the failure of FVs to release VLPs in the absence of the glycoprotein and the accumulation of capsids at the MTOC not being associated with cellular membranes [31,76]. FV particle egress is dependent on a specific interaction between FV Gag and Env involving domains in the N-terminus of both proteins (Gag CC1 and Env LP, see above) [102,103]. This dependency on the specific natural interaction has so far prevented modification of the FV tropism by pseudotyping of FV vector particles with heterologous glycoproteins [110]. FV particles can be engineered to release VLPs in an Env-independent fashion by artificial N-terminal fusion of heterologous membrane targeting signals to Gag [74,84]. However, these constructs remain non-infectious upon coexpression of heterologous glycoproteins or even the cognate Env protein [74,84].

Another unusual feature of FVs, at least in the primate FVs, is the cellular location of their budding, which seems to occur predominantly at intracellular membranes, but to a limited extent as well as at the plasma membrane. Early reports indicated a budding of FVs capsids into the ER, which fitted quite well with the later characterization of a C-terminal KKXX ER-retrieval signal in the FV Env protein of many FV species [23,113]. However, this ER retrieval signal proved to be dispensable for viral replication and only marginal changes in the location of budding could be observed [108]. Furthermore, some non-primate FVs like EFV or FFV naturally lack an ER-retrieval signal and budding of EFV exclusively at the plasma membrane has been described [114,115]. The nature of the vacuolar structures with prominent FV budding structures frequently observed in primate FV expressing adherent cells still awaits further characterization. Due to the high cytopathic effects of FVs *in vitro* and the associated disturbance of intracellular organization this apparently intracellular budding compartment might potentially represent other organelles such as the Golgi or, potentially, plasma membrane invaginations.

The initiation of reverse transcription of the packaged viral RNA genome during or soon after capsid assembly in the virus-producing cell is another unique feature of FVs among retroviruses [25]. Up to 20% of the viral nucleic acids in extracellular FV particles represents DNA, whereas it has been reported to be below 0.001% for orthoretroviruses [26,27]. Intraparticular reverse transcription of the packaged viral RNA genome is not dependent on particle release, since reverse transcription is easily detectable in intracellular accumulated capsids of Env-deficient viruses [25]. In contrast, a proper microenvironment such as regular capsid morphology and yet unidentified cellular requirements, are essential for this process [78,81]. Experiments with RT inhibitors have demonstrated that the infectious viral genome of FVs and FV vectors is mainly DNA [25,27]. However, there are indications that further reverse transcription takes place upon FV target cell entry in a classical orthoretroviral fashion that might contribute to productive infection under certain conditions for example at low MOI [116,117]. In addition, the characterization of FV sequences from feces of wild chimpanzees revealed only RNA genomes and no virion DNA [118]. This may indicate that there are differences between the replicative behavior of FV in cell culture and *in vivo*. However, it is neither known whether the feces-associated FV represent the infectious form of the virus nor what cells in the body these viruses produced.

The feature of late reverse transcription in the FV replication cycle, resulting in potential infectious capsids in the virus producing cell, underlies another unique retroviral feature, the intracellular retrotransposition (IRT) of FV genomes within an infected cell. A reintegration of reverse transcribed FV genomes, reminiscent of hepadnavirus nuclear genome reshuttling, was reported [28]. However, the frequency of 5% reported for cells transfected with Env-deficient PFV vector constructs represents quite an artificial condition, since it leads to an intracellular accumulation of FV capsids [28,29]. Furthermore, not all FVs seem to share this feature, as IRT was not detectable for FFV-derived vectors [27]. Therefore it remains an open question as to whether IRT is an epiphenomenon of env-deleted PFV vectors and occurs upon natural FV infection.

## 9. FV Entry

Little is known about the FV entry process. As previously mentioned, FVs are characterized by having an extremely broad host range, but the potentially ubiquitously expressed FV receptor(s) still awaits its identification [111]. Infection studies using MLV vectors pseudotyped with FV glycoproteins, together with the use of lysosomotropic agents as well as cell-cell fusion assays have been exploited to examine early processes of FV entry post receptor binding [13]. These analyses indicated an uptake of FV particles by endocytosis and showed that FV Env-mediated membrane fusion is a pH-dependent process [13]. However, whereas most FV Env species examined (SFVmac, FFV, BFV, EFV) show a strong induction of glycoprotein fusion activity at low pH, the PFV glycoprotein has quite a high basal fusion activity at neutral pH [13]. Indeed, recent single virus particle tracking studies using autofluorescent protein-tagged FV particles and time-lapse video microscopy support differential uptake routes for PFV and SFVmac [119].

Upon release into the cytoplasm FV capsids are transported on microtubules to the centrosome where they accumulate as intact naked capsids, a process that is inhibited by the microtubule depolimerizing drug Nocodazole [14]. It has been suggested that FV capsids hijack cellular dynein motor protein complexes for this process by a direct interaction of the Gag CC2 domain with the dynein light chain 8 [14]. In G0 arrested cells accumulated FV capsids and viral genomes are detectable at the MTOC for weeks, without any apparent indication for further virus uncoating processes to occur [15]. This is consistent with other studies on the cell-cycle requirements for FV vector transduction, indicating a similar efficiency of FV and HIV-1 vectors in transducing quiescent G0 serum-starved fibroblasts, that was superior to that of MLV vectors [15,18,120]. Only upon cell activation FV disassembly proceeds, resulting in a productive infection [15]. Additional processing of the FV Gag protein by the viral and cellular proteases was suggested to be essential for this process [15,16]. The cellular signals triggering these further uncoating steps remain unclear. For cells arrested in the G1/S phase of the cell cycle, a block in the FV replication cycle has also been reported [121]. In contrast to G0 arrested cells, in this case capsid uncoating upon arrival at the MTOC seems to proceed, as virus genome is detectable in the nucleus [121]. However, under these conditions viral transcription and viral DNA integration is inhibited preventing a productive infection [121]. Thus FV vectors require mitosis for proviral integration and transgene expression [122]. However, they are able to form quite stable transduction intermediates in quiescent cells that can persist in a functional state for very long time periods until the cells reenter the cell cycle.

The integration site profile of different retroviruses has gained increasing interest in recent years as it contributes to their potential to cause cancer in clinical Gene Therapy trials [123,124]. Large-scale studies on FV vector integration site profile revealed that they have a much lower preference for transcription start sites than MLV vectors, which preferentially integrate at these locations [125]. Furthermore, whereas HIV-1 vectors have a preference for integration in transcribed genes this is not the case for FV vectors [126–128]. Thus FV vectors display a more favorable integration site profile than MLV or HIV-1 based vectors. An unusual feature of the FV integrase mediated insertion of the viral genome into the host cell genome is the differential terminal trimming of the linear viral DNA genome [129,130]. Unlike orthoretroviruses that remove usually two nucleotides from the termini of the linear episomal viral reverse transcript, FVs process only the U5 terminus of the 3′ LTR whereas the U3 terminus of the 5′ LTR remains untouched [129,130]. The function of this special mechanistic feature of FV integration is currently unclear.

## 10. FV Vector Systems

During the last 15 years vector systems primarily based on PFV, SFVmac and FFV have been developed that allow production of high titer vector supernatants and efficient transduction of a large variety of target cells [111]. Unlike orthoretroviral vectors, the organization of *cis*-acting sequences (CAS) required for RNA and Pol packaging is more complex [131–133].

**Figure 4:**
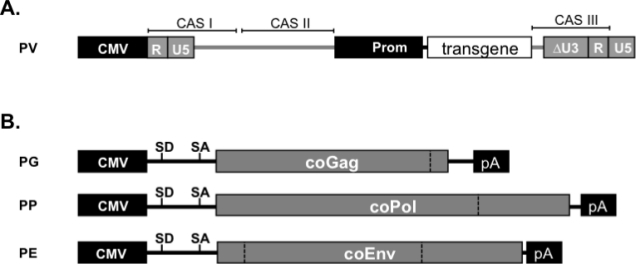
Third generation Prototype FV (PFV) vector system. Schematic outline of the third generation (**A**) transfer vector PV and (**B**) packaging expression vectors for codon-optimized (co) Gag (PG), coPol (PP), and coEnv (PE). CMV: cytomegalovirus immediate early promoter; R: LTR repeat region; U5: LTR unique 5′ region; ΔU3: enhancer-promoter deleted LTR unique 3′ region; Prom: internal heterologous promoter; CAS: *cis*-acting sequence; SD: splice donor; SA: splice acceptor.

The latest generations of PFV transfer vectors are of the self-inactivating-type with minimal CAS elements [61,65,134]. Vector genome transcription is driven by chimeric 5′ LTRs having the U3 region replaced by the strong cytomegalovirus (CMV) immediate early promoter [61,65,134]. FV CAS elements essential for efficient gene transfer comprise three regions of the genome (Figures 2 and 4). CAS-I spans the 5′ LTR R region up to the first 200 nucleotides of the original Gag ORF (Figure 2). It includes the primer binding site (PBS) just downstream of the 5′ LTR U5 region that is complementary to the 3′ end of tRNA^Lys1,2^ used by PFV for initiation of—strand DNA synthesis during reverse transcription [135]. Mutations have been introduced into CAS-I to inactivate the authentic Gag translation start, thereby preventing expression of residual C-terminally truncated Gag peptide sequences [65]. CAS-II was originally identified to be about 2 kb in size and located in the 3′ region of the *pol* ORF and including a central poly-purine tract (PPT) [131–133]. It has been further minimized and is composed of a discontinuous element of 1.2 kb in size harboring RNA packaging as well as proposed Pol encapsidation and central PPT sequences (Figure 2) [61,62]. Finally, CAS-III includes about 40 nucleotides upstream of the 3′ LTR containing the 3′ PPT, about 200 bp of the 5′ end of its U3 region and the complete R region (Figure 4). Although, the FV LTR promoter is virtually inactive in the absence of the viral transactivator Tas, for safety reasons the 3′ LTRs in FV vectors have large deletions in the U3 region encompassing viral promoter and enhancer elements [65,134]. Since transgene expression in FV vectors is not driven by the viral LTR, as it is for example in some murine leukemia virus (MLV)-based vectors, a transgene expression cassette with heterologous promoter is used. Generally this is inserted between CAS-II and CAS-III [65].

State of the art FV packaging systems are composed of three separate expression vectors; one for the viral Gag protein, one for Pol and one for Env (Figure 4). Expression of the original *gag* ORF is strictly dependent on an upstream SD site of FV or heterologous origin [65]. The use of a spliced transcript for *pol* gene expression is also highly recommended. Instead *env* can be expressed from unspliced mRNAs, although expression levels by vectors containing an upstream intron tend to be significantly higher. Separate *gag* and *pol* expression vectors result in higher titers than constructs containing both overlapping ORFs and using the natural *pol* SA in the *gag* ORF for translation of Pol from a spliced RNA [65]. Quite recently, further improvement in vector titers was achieved by expression-optimization of all packaging constructs [81], allowing production of vector supernatants with up to 1 × 10^7^ ffU/mL by transient transfection without further concentration steps, provided that the relative amounts of the packaging plasmids have been adjusted properly [136].

## 11. Experimental FV Vector Applications

FV vectors were shown to be an efficient gene delivery vehicle for different scientific and therapeutic approaches in a variety of different target cells. This includes their ability to transduce efficiently neuronal progenitor cells or embryonic stem cells [137,138]. However, the best-studied application of FV vectors is for gene transfer into hematopoietic stem cells (HSCs) of different origin [139–142]. For example, FV vectors have been demonstrated to transduce efficiently mouse HSCs that are capable of long-term repopulation after consecutive BM transplantation [141,143]. Furthermore, marking experiments of dog HSCs indicate that they have a similar gene transfer potential as HIV-1 vectors [140,144]. Similarly, FV vectors have a proven potential for efficient transduction of human HSC in the xenogenic NOD/SCID mouse model [139,141]. A direct comparison with HIV-1- and MLV- based vectors revealed that they are capable of a similar, if not higher, gene transfer efficiency as HIV-1 vectors and are superior to MLV-based vectors [139]. More importantly, only very short *ex vivo* transduction protocols are required for efficient transduction by FV vectors, which is beneficial for the maintenance of the engraftment potential of the modified HSCs [139,143].

FV were shown not only to enable efficient marker gene transfer, but also transfer of therapeutic genetic material. In a dog model for leukocyte deficiency, transduction of CD34+ BM cells with a correct copy of the CD18 gene followed by transplantation of these modified cells was able to cure the disease phenotype in several animals [145]. In these dogs a long-term stable multilineage marking of the hematopoietic system was observed. Similarly, in a mouse model of Fanconi anemia C a short-term transduction of HSC from Fancc knock-out mice with a Fancc transgene-expressing FV vector reversed their repopulation defect [143].

## 12. Pseudotyping of Orthoretroviral Vectors with FV Env

While pseudotyping of unmodified FV capsids with other than FV glycoproteins is not possible [110], the other way round, *i.e.*, pseudotyping of orthoretroviral capsids with FV Env, works well [146]. Long before the particular topology of FV Env was known [103], attempts to increase the principle possibility of pseudotyping MLV-based vectors by modifying the membrane-spanning-domain of FV TM were undertaken, and led only to a moderate increase in performance of the former [147]. After the discovery of the special characteristics of FV Env, mutants could be generated that allowed for pseudotyping that was as good as, if not superior to, the widely used VSV-G, particularly in transducing HSCs [136,146]. Given the opportunity to concentrate vectors bearing the FV Env [148], this opens up completely new avenues in generating lentiviral (HIV-1) vectors, since the FV Env appears to be less toxic than VSV-G and easier to handle in the vector production process [136,149].

## 13. Safety of FV Vectors

Besides their high efficiency in transducing certain and—from a practical point of view—interesting primary cell populations, such as HSCs, investigations into the safety profile of FV vectors were of prime interest. One of the great advantages of these vectors consists in the fact that they were derived from probably evolutionary extremely old and genomically hardly changed wildtype viruses, which co-evolved with their respective hosts [1]. Upon natural, experimental or accidental infection—including transmission to humans—these viruses have been shown to be apathogenic in contrast to MLV and HIV-1, which induce severe specific diseases [150]. A report on the induction of a subclinical condition in male cats upon FFV infection has, so far, only proved to be anecdotal [151].

The probability of generating replicating virus using modern retroviral vectors—be they derived from gamma-, lenti- or foamy viruses—is so extremely low, that it can be ignored here. Severe side-effects from clinical Gene Therapy trials with retroviral vectors result mainly from two more or less inherent features of the wildtype virus, which are not that easy to alter.

Feature one: Due to the integration profile into the host genome, a given vector may lead to anti-oncogene inactivation or to *proto*-oncogene activation. Generally, the former is regarded less dangerous than the latter. *Proto*-oncogene activation is more likely if the integration of a retroviral vector with its strong enhancers occurs in the vicinity of the cellular gene. However, far-reaching long-distance effects may also contribute. In this respect it is remarkable that the follow-up of individual cellular clones from the FV vector-mediated canine CD18 trial neither revealed malignant transformation nor clonal dominance (*i.e.*, a proliferative *in vivo* advantage of certain cells), even when the vector integrated in the vicinity of growth-promoting genes [152]. This study was performed more than three years following the initial bone marrow transplantation that cured the animals. It remains to be seen whether particular features of the host, of the disease or the vector are responsible for this beneficial outcome. It may turn out that FV LTRs behave like natural insulators, even in their deleted form present in the vector genomes. With respect to the integration profile, FV vectors show a much more random integration than their gamma- and lentiviral relatives, although this is not completely random [126,128,145].

Feature two: The other mechanism by which an integrating vector may harm the host cell is by readthrough of transcripts generated by the vector into cellular *proto*-oncogenes. Here the strength of the polyA signal in the 3′ LTR appears to play a crucial role. This is not easy to determine experimentally, but a system has been devised recently and the analysis revealed the following order: gammaretroviruses << lentiviruses < foamy viruses [153]. Also in this respect it appears that FV vectors take up a unique position among retroviral vectors that warrant further investigation and exploitation.

As far as non-integrating FV vectors in generating induced pluripotent stem-cells (iPSC) are concerned [154], only time will tell whether they really offer an advantage over other means of viral or non-viral gene transfer [155].

## 14. Conclusions and Outlook

Vectors derived from FV have some features with respect to efficiency and safety which make them at least as good as their orthoretroviral cousins. These features appear to be a direct consequence of the particular FV replication strategy. Therefore, the analysis of any aspect of this replication strategy will be beneficial to the improvement of current FV vector systems. In the light of what has been detailed above, CD18 deficiency and Fanconi anemia are probably the first human diseases for which a clinical trial using FV vectors will be performed.

## References

[1] Switzer WM, Salemi M, Shanmugam V, Gao F, Cong ME, Kuiken C, Bhullar V, Beer BE, Vallet D, Gautier-Hion A (2005). Ancient co-speciation of simian foamy viruses and primates. Nature.

[2] Saib A (2003). Non-primate foamy viruses. Curr Top Microbiol Immunol.

[3] Switzer WM, Bhullar V, Shanmugam V, Cong ME, Parekh B, Lerche NW, Yee JL, Ely JJ, Boneva R, Chapman LE (2004). Frequent simian foamy virus infection in persons occupationally exposed to nonhuman primates. J Virol.

[4] Heneine W, Schweizer M, Sandstrom P, Folks T (2003). Human infection with foamy viruses. Curr Top Microbiol Immunol.

[5] Delelis O, Lehmann-Che J, Saib A (2004). Foamy viruses—A world apart. Curr Opin Microbiol.

[6] Linial ML, Fan H, Hahn B, Löwer R, Neil J, Quackenbush S, Rethwilm A, Sonigo P, Stoye JP, Tristem M, Fauquet CM, Mayo MA, Maniloff J, Desselberger U, Ball LA (2005). Retroviridae. Virus Taxonomy.

[7] Achong BG, Mansell PWA, Epstein MA, Clifford P (1971). An unusual virus in cultures from a human nasopharyngeal carcinoma. J Natl Cancer Inst.

[8] Herchenröder O, Renne R, Loncar D, Cobb EK, Murthy KK, Schneider J, Mergia A, Luciw PA (1994). Isolation, cloning, and sequencing of simian foamy viruses from chimpanzees (SFVcpz): High homology to human foamy virus (HFV). Virology.

[9] Schweizer M, Turek R, Hahn H, Schliephake A, Netzer KO, Eder G, Reinhardt M, Rethwilm A, Neumann-Haefelin D (1995). Markers of foamy virus infections in monkeys, apes, and accidentally infected humans: Appropriate testing fails to confirm suspected foamy virus prevalence in humans. AIDS Res Hum Retroviruses.

[10] Schweizer M, Turek R, Reinhardt M, Neumann-Haefelin D (1994). Absence of foamy virus DNA in Graves’ disease. AIDS Res Hum Retroviruses.

[11] Epstein MA (2004). Simian retroviral infectins in human beings. Lancet (Correspondence).

[12] Jones-Engel L, May CC, Engel GA, Steinkraus KA, Schillaci MA, Fuentes A, Rompis A, Chalise MK, Aggimarangsee N, Feeroz MM (2008). Diverse contexts of zoonotic transmission of simian foamy viruses in Asia. Emerg Infect Dis.

[13] Picard-Maureau M, Jarmy G, Berg A, Rethwilm A, Lindemann D (2003). Foamy virus envelope glycoprotein-mediated entry involves a pH-dependent fusion process. J Virol.

[14] Petit C, Giron ML, Tobaly-Tapiero J, Bittoun P, Real E, Jacob Y, Tordo N, De The H, Saib A (2003). Targeting of incoming retroviral Gag to the centrosome involves a direct interaction with the dynein light chain 8. J Cell Sci.

[15] Lehmann-Che J, Renault N, Giron ML, Roingeard P, Clave E, Tobaly-Tapiero J, Bittoun P, Toubert A, de The H, Saib A (2007). Centrosomal latency of incoming foamy viruses in resting cells. PLoS Pathog.

[16] Lehmann-Che J, Giron ML, Delelis O, Lochelt M, Bittoun P, Tobaly-Tapiero J, de The H, Saib A (2005). Protease-dependent uncoating of a complex retrovirus. J Virol.

[17] Patton GS, Erlwein O, McClure MO (2004). Cell-cycle dependence of foamy virus vectors. J Gen Virol.

[18] Trobridge G, Russell DW (2004). Cell cycle requirements for transduction by foamy virus vectors compared to those of oncovirus and lentivirus vectors. J Virol.

[19] Tobaly-Tapiero J, Bittoun P, Lehmann-Che J, Delelis O, Giron ML, de The H, Saib A (2008). Chromatin tethering of incoming foamy virus by the structural Gag protein. Traffic.

[20] Löchelt M (2003). Foamy virus transactivation and gene expression. Curr Top Microbiol Immunol.

[21] Bodem J, Schied T, Gabriel R, Rammling M, Rethwilm A (2011). Foamy virus nuclear RNA export is distinct from that of other retroviruses. J Virol.

[22] Renault N, Tobaly-Tapiero J, Paris J, Giron ML, Coiffic A, Roingeard P, Saib A (2011). A nuclear export signal within the structural Gag protein is required for prototype foamy virus replication. Retrovirology.

[23] Goepfert PA, Shaw KL, Ritter GD, Mulligan MJ (1997). A sorting motif localizes the foamy virus glycoprotein to the endoplasmic reticulum. J Virol.

[24] Stanke N, Stange A, Lüftenegger D, Zentgraf H, Lindemann D (2005). Ubiquitination of the prototype foamy virus envelope glycoprotein leader peptide regulates subviral particle release. J Virol.

[25] Moebes A, Enssle J, Bieniasz PD, Heinkelein M, Lindemann D, Bock M, McClure MO, Rethwilm A (1997). Human foamy virus reverse transcription that occurs late in the viral replication cycle. J Virol.

[26] Yu SF, Sullivan MD, Linial ML (1999). Evidence that the human foamy virus genome is DNA. J Virol.

[27] Roy J, Rudolph W, Juretzek T, Gartner K, Bock M, Herchenroder O, Lindemann D, Heinkelein M, Rethwilm A (2003). Feline foamy virus genome and replication strategy. J Virol.

[28] Heinkelein M, Pietschmann T, Jarmy G, Dressler M, Imrich H, Thurow J, Lindemann D, Bock M, Moebes A, Roy J (2000). Efficient intracellular retrotransposition of an exogenous primate retrovirus genome. EMBO J.

[29] Heinkelein M, Rammling M, Juretzek T, Lindemann D, Rethwilm A (2003). Retrotransposition and cell-to-cell transfer of foamy viruses. J Virol.

[30] Baldwin DN, Linial ML (1998). The roles of Pol and Env in the assembly pathway of human foamy virus. J Virol.

[31] Fischer N, Heinkelein M, Lindemann D, Enssle J, Baum C, Werder E, Zentgraf H, Muller JG, Rethwilm A (1998). Foamy virus particle formation. J Virol.

[32] Patton GS, Morris SA, Chung W, Bieniasz PD, McClure MO (2005). Identification of domains in gag important for prototypic foamy virus egress. J Virol.

[33] Stange A, Mannigel I, Peters K, Heinkelein M, Stanke N, Cartellieri M, Gottlinger H, Rethwilm A, Zentgraf H, Lindemann D (2005). Characterization of prototype foamy virus gag late assembly domain motifs and their role in particle egress and infectivity. J Virol.

[34] Löchelt M, Muranyi W, Flügel RM (1993). Human foamy virus genome possesses an internal, Bel-1-dependent and functional promoter. Proc Natl Acad Sci U S A.

[35] Löchelt M, Flugel RM, Aboud M (1994). The human foamy virus internal promoter directs the expression of the functional Bel 1 transactivator and Bet protein early after infection. J Virol.

[36] Löchelt M, Yu SF, Linial ML, Flügel RM (1995). The human foamy virus internal promoter is required for efficient gene expression and infectivity. Virology.

[37] Rethwilm A, Erlwein O, Baunach G, Maurer B, ter Meulen V (1991). The transcriptional transactivator of human foamy virus maps to the bel 1 genomic region. Proc Natl Acad Sci U S A.

[38] Keller A, Partin KM, Löchelt M, Bannert H, Flügel RM, Cullen BR (1991). Characterization of the transcriptional trans activator of human foamy retrovirus. J Virol.

[39] Kang Y, Blair WS, Cullen BR (1998). Identification and functional characterization of a high-affinity Bel-1 DNA binding site located in the human foamy virus internal promoter. J Virol.

[40] He F, Blair WS, Fukushima J, Cullen BR (1996). The human foamy virus Bel-1 transcription factor is a sequence-specific DNA binding protein. J Virol.

[41] Wang J, Tan J, Guo H, Zhang Q, Jia R, Xu X, Geng Y, Qiao W (2010). Bovine foamy virus transactivator BTas interacts with cellular RelB to enhance viral transcription. J Virol.

[42] Wang J, Tan J, Zhang X, Guo H, Zhang Q, Guo T, Geng Y, Qiao W (2010). BFV activates the NF-kappaB pathway through its transactivator (BTas) to enhance viral transcription. Virology.

[43] Tan J, Hao P, Jia R, Yang W, Liu R, Wang J, Xi Z, Geng Y, Qiao W (2010). Identification and functional characterization of BTas transactivator as a DNA-binding protein. Virology.

[44] Baunach G, Maurer B, Hahn H, Kranz M, Rethwilm A (1993). Functional analysis of human foamy virus accessory reading frames. J Virol.

[45] Muranyi W, Flugel RM (1991). Analysis of splicing patterns of human spumaretrovirus by polymerase chain reaction reveals complex RNA structures. J Virol.

[46] Schmidt M, Rethwilm A (1995). Replicating foamy virus-based vectors directing high level expression of foreign genes. Virology.

[47] Löchelt M, Romen F, Bastone P, Muckenfuss H, Kirchner N, Kim YB, Truyen U, Rosler U, Battenberg M, Saib A (2005). The antiretroviral activity of APOBEC3 is inhibited by the foamy virus accessory Bet protein. Proc Natl Acad Sci U S A.

[48] Russell RA, Wiegand HL, Moore MD, Schafer A, McClure MO, Cullen BR (2005). Foamy virus Bet proteins function as novel inhibitors of the APOBEC3 family of innate antiretroviral defense factors. J Virol.

[49] Perkovic M, Schmidt S, Marino D, Russell RA, Stauch B, Hofmann H, Kopietz F, Kloke BP, Zielonka J, Strover H (2009). Species-specific inhibition of APOBEC3C by the prototype foamy virus protein bet. J Biol Chem.

[50] Cullen BR (2002). Using retroviruses to study the nuclear export of mRNA. Results Probl Cell Differ.

[51] Cullen BR (2003). Nuclear RNA export. J Cell Sci.

[52] Gruter P, Tabernero C, von Kobbe C, Schmitt C, Saavedra C, Bachi A, Wilm M, Felber BK, Izaurralde E (1998). TAP, the human homolog of Mex67p, mediates CTE-dependent RNA export from the nucleus. Mol Cell.

[53] Zolotukhin AS, Michalowski D, Smulevitch S, Felber BK (2001). Retroviral constitutive transport element evolved from cellular TAP(NXF1)-binding sequences. J Virol.

[54] Yu SF, Baldwin DN, Gwynn SR, Yendapalli S, Linial ML (1996). Human foamy virus replication: a pathway distinct from that of retroviruses and hepadnaviruses. Science.

[55] Bodem J, Lochelt M, Winkler I, Flower RP, Delius H, Flügel RM (1996). Characterization of the spliced pol transcript of feline foamy virus: the splice acceptor site of the pol transcript is located in gag of foamy viruses. J Virol.

[56] Jordan I, Enssle J, Guttler E, Mauer B, Rethwilm A (1996). Expression of human foamy virus reverse transcriptase involves a spliced pol mRNA. Virology.

[57] Enssle J, Jordan I, Mauer B, Rethwilm A (1996). Foamy virus reverse transcriptase is expressed independently from the Gag protein. Proc Natl Acad Sci U S A.

[58] Lee EG, Kuppers D, Horn M, Roy J, May C, Linial ML (2008). A premature termination codon mutation at the C terminus of foamy virus Gag downregulates the levels of spliced pol mRNA. J Virol.

[59] Swiersy A, Wiek C, Reh J, Zentgraf H, Lindemann D (2011). Orthoretroviral-like Prototype Foamy Virus Gag-Pol Expression is Compatible with Viral Replication. Retrovirology.

[60] Heinkelein M, Leurs C, Rammling M, Peters K, Hanenberg H, Rethwilm A (2002). Pregenomic RNA is required for efficient incorporation of pol polyprotein into foamy virus capsids. J Virol.

[61] Wiktorowicz T, Peters K, Armbruster N, Steinert AF, Rethwilm A (2009). Generation of an improved foamy virus vector by dissection of cis-acting sequences. J Gen Virol.

[62] Peters K, Wiktorowicz T, Heinkelein M, Rethwilm A (2005). RNA and protein requirements for incorporation of the pol protein into foamy virus particles. J Virol.

[63] Lee EG, Linial ML (2008). The C terminus of foamy retrovirus Gag contains determinants for encapsidation of Pol protein into virions. J Virol.

[64] Roy J, Linial ML (2007). Role of the foamy virus pol cleavage site in viral replication. J Virol.

[65] Heinkelein M, Dressler M, Jarmy G, Rammling M, Imrich H, Thurow J, Lindemann D, Rethwilm A (2002). Improved primate foamy virus vectors and packaging constructs. J Virol.

[66] Heinkelein M, Thurow J, Dressler M, Imrich H, Neumann-Haefelin D, McClure MO, Rethwilm A (2000). Complex effects of deletions in the 5′ untranslated region of primate foamy virus on viral gene expression and RNA packaging. J Virol.

[67] Flügel RM, Pfrepper KI (2003). Proteolytic processing of foamy virus Gag and Pol proteins. Curr Top Microbiol Immunol.

[68] Cartellieri M, Rudolph W, Herchenröder O, Lindemann D, Rethwilm A (2005). Determination of the relative amounts of Gag and Pol proteins in foamy virus particles. Retrovirology.

[69] Enssle J, Fischer N, Moebes A, Mauer B, Smola U, Rethwilm A (1997). Carboxy-terminal cleavage of the human foamy virus Gag precursor molecule is an essential step in the viral life cycle. J Virol.

[70] Zemba M, Wilk T, Rutten T, Wagner A, Flügel RM, Löchelt M (1998). The carboxy-terminal p3Gag domain of the human foamy virus Gag precursor is required for efficient virus infectivity. Virology.

[71] Giron ML, Colas S, Wybier J, Rozain F, Emanoil-Ravier R (1997). Expression and maturation of human foamy virus Gag precursor polypeptides. J Virol.

[72] Matthes D, Wiktorowicz T, Zahn J, Bodem J, Stanke N, Lindemann D, Rethwilm A (2011). Basic Residues in the Foamy Virus Gag Protein. J Virol.

[73] Tobaly-Tapiero J, Bittoun P, Giron ML, Neves M, Koken M, Saib A, de The H (2001). Human foamy virus capsid formation requires an interaction domain in the N terminus of Gag. J Virol.

[74] Eastman SW, Linial ML (2001). Identification of a conserved residue of foamy virus Gag required for intracellular capsid assembly. J Virol.

[75] Cartellieri M, Herchenröder O, Rudolph W, Heinkelein M, Lindemann D, Zentgraf H, Rethwilm A (2005). N-terminal Gag domain required for foamy virus particle assembly and export. J Virol.

[76] Yu SF, Eastman SW, Linial ML (2006). Foamy virus capsid assembly occurs at a pericentriolar region through a cytoplasmic targeting/retention signal in Gag. Traffic.

[77] Rhee SS, Hunter E (1990). A single amino acid substitution within the matrix protein of a type D retrovirus converts its morphogenesis to that of a type C retrovirus. Cell.

[78] Mannigel I, Stange A, Zentgraf H, Lindemann D (2007). Correct capsid assembly mediated by a conserved YXXLGL motif in prototype foamy virus Gag is essential for infectivity and reverse transcription of the viral genome. J Virol.

[79] Swanstrom R, Wills JW, Coffin JM, Hughes SH, Varmus HE (1997). Synthesis, Assembly, and Processing of Viral Proteins. Retroviruses.

[80] Schliephake AW, Rethwilm A (1994). Nuclear localization of foamy virus Gag precursor protein. J Virol.

[81] Müllers E, Uhlig T, Stirnnagel K, Fiebig U, Zentgraf H, Lindemann D (2011). Novel functions of Prototype Foamy Virus Gag GR boxes in reverse transcription and particle morphogenesis. J Virol.

[82] Yu SF, Edelmann K, Strong RK, Moebes A, Rethwilm A, Linial ML (1996). The carboxyl terminus of the human foamy virus Gag protein contains separable nucleic acid binding and nuclear transport domains. J Virol.

[83] Bodem J, Zemba M, Flügel RM (1998). Nuclear localization of the functional Bel 1 transactivator but not of the gag proteins of the feline foamy virus. Virology.

[84] Zhadina M, McClure MO, Johnson MC, Bieniasz PD (2007). Ubiquitin-dependent virus particle budding without viral protein ubiquitination. Proc Natl Acad Sci U S A.

[85] Imrich H, Heinkelein M, Herchenröder O, Rethwilm A (2000). Primate foamy virus Pol proteins are imported into the nucleus. J Gen Virol.

[86] An DG, Hyun U, Shin CG (2008). Characterization of nuclear localization signals of the prototype foamy virus integrase. J Gen Virol.

[87] Hartl MJ, Mayr F, Rethwilm A, Wohrl BM (2010). Biophysical and enzymatic properties of the simian and prototype foamy virus reverse transcriptases. Retrovirology.

[88] Rinke CS, Boyer PL, Sullivan MD, Hughes SH, Linial ML (2002). Mutation of the catalytic domain of the foamy virus reverse transcriptase leads to loss of processivity and infectivity. J Virol.

[89] Boyer PL, Stenbak CR, Clark PK, Linial ML, Hughes SH (2004). Characterization of the polymerase and RNase H activities of human foamy virus reverse transcriptase. J Virol.

[90] Boyer PL, Stenbak CR, Hoberman D, Linial ML, Hughes SH (2007). In vitro fidelity of the prototype primate foamy virus (PFV) RT compared to HIV-1 RT. Virology.

[91] Gärtner K, Wiktorowicz T, Park J, Mergia A, Rethwilm A, Scheller C (2009). Accuracy estimation of foamy virus genome copying. Retrovirology.

[92] Hartl MJ, Schweimer K, Reger MH, Schwarzinger S, Bodem J, Rosch P, Wohrl BM (2010). Formation of transient dimers by a retroviral protease. Biochem J.

[93] Hartl MJ, Wohrl BM, Rosch P, Schweimer K (2008). The solution structure of the simian foamy virus protease reveals a monomeric protein. J Mol Biol.

[94] Lee EG, Roy J, Jackson D, Clark P, Boyer PL, Hughes SH, Linial M (2011). Foamy retrovirus integrase contains a Pol dimerization domain required for protease activation. J Virol.

[95] Hartl MJ, Bodem J, Jochheim F, Rethwilm A, Rösch P, Wöhrl BM (2011). Regulation of foamy virus protease activity by viral RNA—A novel and unique mechanism among retroviruses. J Virol.

[96] Hare S, Gupta SS, Valkov E, Engelman A, Cherepanov P (2010). Retroviral intasome assembly and inhibition of DNA strand transfer. Nature.

[97] Cherepanov P (2010). Integrase iluminated. EMBO Rep.

[98] Cherepanov P, Maertens GN, Hare S (2011). Structural insights into the retroviral DNA integration apperatus. Curr Opin Struct Bio.

[99] Hare S, Vos AM, Clayton RR, Thuring JW, Cummings MD, Cherepanov P (2010). Molecular mechanisms of retroviral integrase inhibition and the evolution of viral resistance. Proc Natl Acad Sci U S A.

[100] Maertens GN, Hare S, Cherepanov P (2010). The mechanism of retroviral integration from X-ray structures of its key intermediates. Nature.

[101] Krishnan L, Li X, Naraharisetty HL, Hare S, Cherepanov P, Engelman A (2010). Structure-based modeling of the functional HIV-1 intasome and its inhibition. Proc Natl Acad Sci U S A.

[102] Wilk T, Geiselhart V, Frech M, Fuller SD, Flügel RM, Löchelt M (2001). Specific interaction of a novel foamy virus Env leader protein with the N-terminal Gag domain. J Virol.

[103] Lindemann D, Pietschmann T, Picard-Maureau M, Berg A, Heinkelein M, Thurow J, Knaus P, Zentgraf H, Rethwilm A (2001). A particle-associated glycoprotein signal peptide essential for virus maturation and infectivity. J Virol.

[104] Duda A, Stange A, Luftenegger D, Stanke N, Westphal D, Pietschmann T, Eastman SW, Linial ML, Rethwilm A, Lindemann D (2004). Prototype foamy virus envelope glycoprotein leader peptide processing is mediated by a furin-like cellular protease, but cleavage is not essential for viral infectivity. J Virol.

[105] Geiselhart V, Bastone P, Kempf T, Schnolzer M, Löchelt M (2004). Furin-mediated cleavage of the feline foamy virus Env leader protein. J Virol.

[106] Luftenegger D, Picard-Maureau M, Stanke N, Rethwilm A, Lindemann D (2005). Analysis and function of prototype foamy virus envelope N glycosylation. J Virol.

[107] Geiselhart V, Schwantes A, Bastone P, Frech M, Löchelt M (2003). Features of the Env leader protein and the N-terminal Gag domain of feline foamy virus important for virus morphogenesis. Virology.

[108] Goepfert PA, Shaw K, Wang G, Bansal A, Edwards BH, Mulligan MJ (1999). An endoplasmic reticulum retrieval signal partitions human foamy virus maturation to intracytoplasmic membranes. J Virol.

[109] Stange A, Lüftenegger D, Reh J, Weissenhorn W, Lindemann D (2008). Subviral particle release determinants of prototype foamy virus. J Virol.

[110] Pietschmann T, Heinkelein M, Heldmann M, Zentgraf H, Rethwilm A, Lindemann D (1999). Foamy virus capsids require the cognate envelope protein for particle export. J Virol.

[111] Hill CL, Bieniasz PD, McClure MO (1999). Properties of human foamy virus relevant to its development as a vector for gene therapy. J Gen Virol.

[112] Stirnnagel K, Lüftenegger D, Stange A, Swiersy A, Mullers E, Reh J, Stanke N, Grosse A, Chiantia S, Keller H (2010). Analysis of prototype foamy virus particle-host cell interaction with autofluorescent retroviral particles. Retrovirology.

[113] Goepfert PA, Wang G, Mulligan MJ (1995). Identification of an ER retrieval signal in a retroviral glycoprotein. Cell.

[114] Winkler I, Bodem J, Haas L, Zemba M, Delius H, Flower R, Flügel RM, Löchelt M (1997). Characterization of the genome of feline foamy virus and its proteins shows distinct features different from those of primate spumaviruses. J Virol.

[115] Tobaly-Tapiero J, Bittoun P, Neves M, Guillemin MC, Lecellier CH, Puvion-Dutilleul F, Gicquel B, Zientara S, Giron ML, de The H (2000). Isolation and characterization of an equine foamy virus. J Virol.

[116] Zamborlini A, Renault N, Saib A, Delelis O (2010). Early reverse transcription is essential for productive foamy virus infection. PLoS ONE.

[117] Delelis O, Saib A, Sonigo P (2003). Biphasic DNA synthesis in spumaviruses. J Virol.

[118] Liu W, Worobey M, Li Y, Keele BF, Bibollet-Ruche F, Guo Y, Goepfert PA, Santiago ML, Ndjango JB, Neel C (2008). Molecular ecology and natural history of simian foamy virus infection in wild-living chimpanzees. PLoS Pathog.

[119] Stirnnagel K, Dupont A, Schupp D, Perrotton F, Müllers E, Lindemann D, Lamb DC (2011). Insights into uptake and fusion strategies exploited by a non-conventional retrovirus.

[120] Russell DW, Miller AD (1996). Foamy virus vectors. J Virol.

[121] Lo YT, Tian T, Nadeau PE, Park J, Mergia A (2010). The foamy virus genome remains unintegrated in the nuclei of G1/S phase-arrested cells, and integrase is critical for preintegration complex transport into the nucleus. J Virol.

[122] Bieniasz PD, Weiss RA, McClure MO (1995). Cell cycle dependence of foamy retrovirus infection. J Virol.

[123] Hacein-Bey-Abina S, Garrigue A, Wang G, Soulier J, Lim A, Morillon E, Clappier E, Caccavelli L, Delabesse E, Beldjord K (2008). Insertional oncogenesis in 4 patients after retrovirus-mediated gene therapy of SCID-X1. J Clin Invest.

[124] Maetzig T, Galla M, Baum C, Schambach A (2011). Gammaretroviral Vectors: Biology, Technology and Application. Viruses.

[125] Wu X, Li Y, Crise B, Burgess SM (2003). Transcription start regions in the human genome are favored targets for MLV integration. Science.

[126] Trobridge GD, Miller DG, Jacobs MA, Allen JM, Kiem HP, Kaul R, Russell DW (2006). Foamy virus vector integration sites in normal human cells. Proc Natl Acad Sci U S A.

[127] Schröder AR, Shinn P, Chen H, Berry C, Ecker JR, Bushman F (2002). HIV-1 integrationin the human genome favors active genes and local hotspots. Cell.

[128] Nowrouzi A, Dittrich M, Klanke C, Heinkelein M, Rammling M, Dandekar T, von Kalle C, Rethwilm A (2006). Genome-wide mapping of foamy virus vector integrations into a human cell line. J Gen Virol.

[129] Juretzek T, Holm T, Gärtner K, Kanzler S, Lindemann D, Herchenröder O, Picard-Maureau M, Rammling M, Heinkelein M, Rethwilm A (2004). Foamy virus integration. J Virol.

[130] Enssle J, Moebes A, Heinkelein M, Panhuysen M, Mauer B, Schweizer M, Neumann-Haefelin D, Rethwilm A (1999). An active foamy virus integrase is required for virus replication. J Gen Virol.

[131] Heinkelein M, Schmidt M, Fischer N, Moebes A, Lindemann D, Enssle J, Rethwilm A (1998). Characterization of a cis-acting sequence in the Pol region required to transfer human foamy virus vectors. J Virol.

[132] Erlwein O, Bieniasz PD, McClure MO (1998). Sequences in pol are required for transfer of human foamy virus-based vectors. J Virol.

[133] Wu M, Chari S, Yanchis T, Mergia A (1998). cis-Acting sequences required for simian foamy virus type 1 vectors. J Virol.

[134] Trobridge G, Josephson N, Vassilopoulos G, Mac J, Russell DW (2002). Improved foamy virus vectors with minimal viral sequences. Mol Ther.

[135] Maurer B, Bannert H, Darai G, Flügel RM (1988). Analysis of the primary structure of the long terminal repeat and the gag and the pol genes of the human spumaretrovirs. J Virol.

[136] Lindemann D (2011). Technische Universität Dresden, Dresden, Germany.

[137] Rothenaigner I, Kramer S, Meggendorfer M, Rethwilm A, Brack-Werner R (2009). Transduction of human neural progenitor cells with foamy virus vectors for differentiation-dependent gene expression. Gen Ther.

[138] Gharwan H, Hirata RK, Wang P, Richard RE, Wang L, Olson E, Allen J, Ware CB, Russell DW (2007). Transduction of human embryonic stem cells by foamy virus vectors. Mol Ther.

[139] Leurs C, Jansen M, Pollok KE, Heinkelein M, Schmidt M, Wissler M, Lindemann D, Von Kalle C, Rethwilm A, Williams DA (2003). Comparison of three retroviral vector systems for transduction of nonobese diabetic/severe combined immunodeficiency mice repopulating human CD34+ cord blood cells. Hum Gene Ther.

[140] Kiem HP, Allen J, Trobridge G, Olson E, Keyser K, Peterson L, Russell DW (2007). Foamy virus-mediated gene transfer to canine repopulating cells. Blood.

[141] Josephson NC, Trobridge G, Russell DW (2004). Transduction of long-term and mobilized peripheral blood-derived NOD/SCID repopulating cells by foamy virus vectors. Hum Gene Ther.

[142] Hirata RK, Miller AD, Andrews RG, Russell DW (1996). Transduction of hematopoietic cells by foamy virus vectors. Blood.

[143] Si Y, Pulliam AC, Linka Y, Ciccone S, Leurs C, Yuan J, Eckermann O, Fruehauf S, Mooney S, Hanenberg H (2008). Overnight transduction with foamyviral vectors restores the long-term repopulating activity of Fancc-/- stem cells. Blood.

[144] Trobridge GD, Allen JM, Peterson L, Ironside CG, Russell D, Kiem HP (2009). Foamy and Lentiviral Vectors Transduce Canine Long-term Repopulating Cells at Similar Efficiency. Hum Gene Ther.

[145] Bauer TR, Allen JM, Hai M, Tuschong LM, Khan IF, Olson EM, Adler RL, Burkholder TH, Gu YC, Russell D (2008). Successful treatment of canine leukocyte adhesion deficiency by foamy virus vectors. Nat Med.

[146] Wurm M, Schambach A, Lindemann D, Hanenberg H, Standker L, Forssmann WG, Blasczyk R, Horn PA (2010). The influence of semen-derived enhancer of virus infection on the efficiency of retroviral gene transfer. J Gene Med.

[147] Lindemann D, Bock M, Schweizer M, Rethwilm A (1997). Efficient pseudotyping of murine leukemia virus particles with chimeric human foamy virus envelope proteins. J Virol.

[148] Trobridge G, Vassilopoulos G, Josephson N, Russell DW (2002). Gene transfer with foamy virus vectors. Methods Enzymol.

[149] Kühlke K (2011). EUFETS GmbH, Idar-Oberstein, Germany.

[150] Linial M (2000). Why aren’t foamy viruses pathogenic?. Trends Microbiol.

[151] German AC, Harbour DA, Helps CR, Gruffydd-Jones TJ (2008). Is feline foamy virus really apathogenic?. Vet Immunol Immunopathol.

[152] Ohmine K, Li Y, Bauer TR, Hickstein DD, Russell DW (2011). Tracking of Specific Integrant Clones in Dogs Treated with Foamy Virus Vectors. Hum Gene Ther.

[153] Hendrie PC, Huo Y, Stolitenko RB, Russell DW (2008). A rapid and quantitative assay for measuring neighboring gene activation by vector proviruses. Mol Ther.

[154] Deyle DR, Li Y, Olson EM, Russell DW (2010). Nonintegrating foamy virus vectors. J Virol.

[155] Warren L, Manos PD, Ahfeldt T, Loh YH, Li H, Lau F, Ebina W, Mandal PK, Smith ZD, Meissner A (2010). Highly efficient reprogramming to pluripotency and directed differentiation of human cells with synthetic modified mRNA. Cell Stem Cell.

